# Severe *Plasmodium falciparum* infection mimicking acute myocardial infarction

**DOI:** 10.1186/1475-2875-13-341

**Published:** 2014-08-30

**Authors:** Helmi Sulaiman, Muhammad Dzafir Ismail, Maisarah Jalalonmuhali, Nadia Atiya, Sasheela Ponnampalavanar

**Affiliations:** Infectious Disease Unit, Faculty of Medicine, University Malaya, Kuala Lumpur, Malaysia; Cardiology Unit, Faculty of Medicine, University Malaya, Kuala Lumpur, Malaysia; Nephrology Unit, Faculty of Medicine, University Malaya, Kuala Lumpur, Malaysia; Department of Medical Microbiology, Faculty of Medicine, University Malaya, Kuala Lumpur, Malaysia

**Keywords:** Myocardial infarction, Severe *Plasmodium falciparum*, Malaria, Coronary angiography

## Abstract

This case report describes a case of presumed acute myocardial infarction in a returned traveler who was later diagnosed to have severe malaria. Emergency coronary angiography was normal and subsequent peripheral blood film was positive for *Plasmodium falciparum*.

## Background

Severe *Plasmodium falciparum* infection among returned travellers can easily be missed by treating physicians, in the event a detailed travel history is not taken at presentation. The many ways a severe malaria can present further complicates the issue as is illustrated in the case below whereby fever was not a predominant component in the presenting complaint but rather an organ-specific complication that prompted the patient seeking medical attention at emergency department (ED) with severe chest pain. This quickly escalated into a prompt cardiology referral and subsequent invasive angiography following an abnormal electrocardiogram that strongly suggested a case of myocardial infarction. Fortunately, the sole sample sent from the ED for malaria blood film examination proved invaluable in this case, which allowed the targeted treatment for malaria to be instituted 24 hours following presentation.

## Case presentation

In this case, a 51-year old local man presented to the emergency department (ED) with acute onset of central chest pain. The worst intensity was experienced four hours before arrival and was self-graded as 6/10. There was associated profuse sweating. His only cardiovascular disease risk factor was 40**-**pack/year history of smoking. On examination, he was afebrile and his blood pressure and pulse rate were 94/53 mmHg and 125/min, respectively. He was conscious and alert, had mild pallor, icterus, tea-coloured urine and hepatomegaly of three finger-breadths under right hypocondrium with dull Traube’s space. There were no remarkable findings on respiratory and cardiovascular examination.

**Figure 1:**
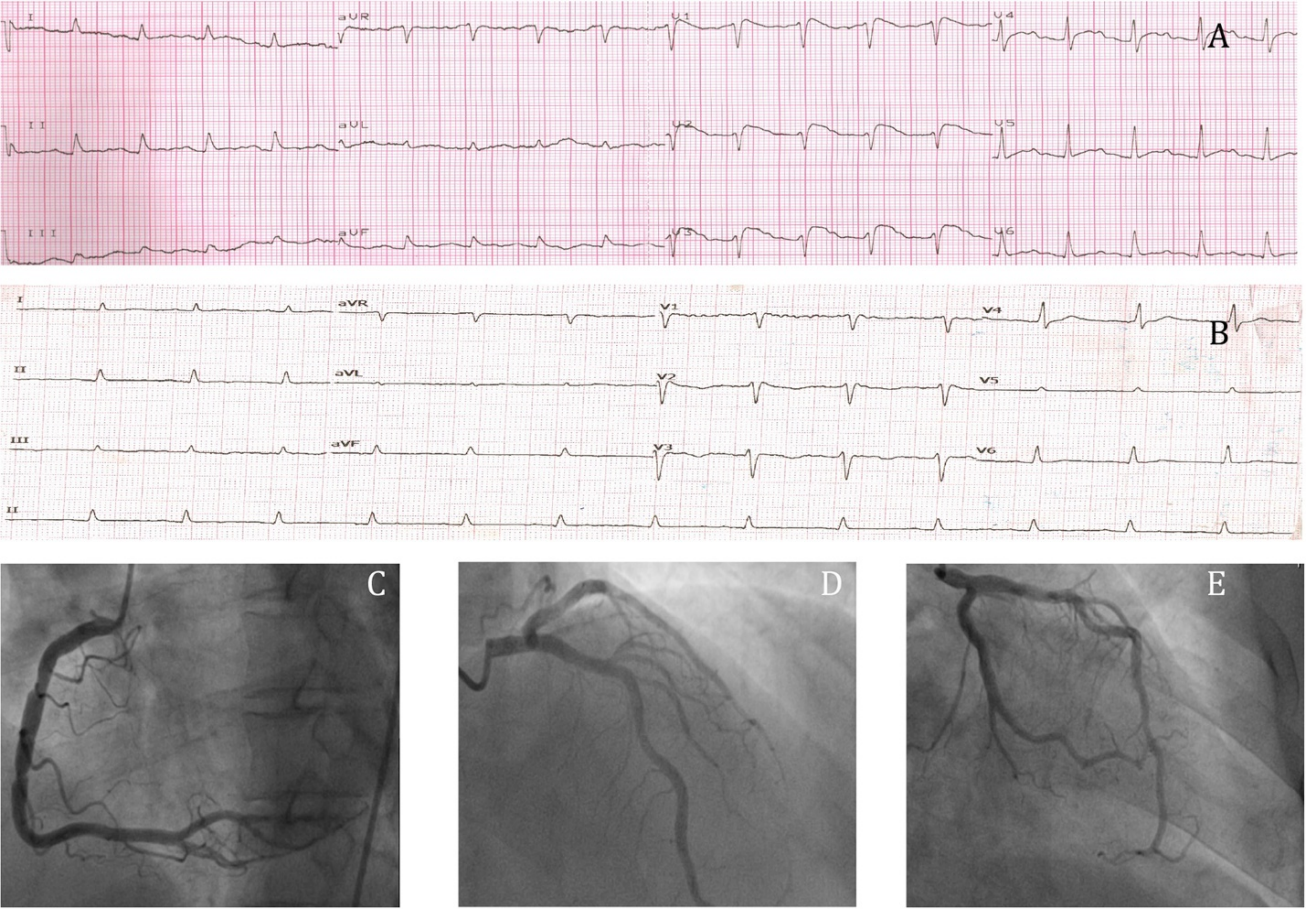
**ECGs and the angiogram studies. A** shows the ECG that was done at presentation, which showed ST elevations on the V1 to V3 leads suggestive of acute anteroseptal myocardial infarction. **B** shows the ECG done 5 days after the first ECG, which showed resolution of ST segment elevations without Q waves development. **C**, **D** and **E** show normal coronary arteries of right coronary artery, left anterior descending artery, left main stem artery and left circumflex artery respectively.

The electrocardiogram (ECG) revealed greater than 2 mm ST segment elevation in leads V1 to V3 suggestive of acute anteroseptal myocardial infarction (AMI) (Figure 1). Cardiac biomarkers inclusive of troponin I, creatinine kinase and creatinine kinase MB were normal (CK, 105 U/L; CKMB was less than 1 ng/mL; trop I was less than 0.01 ng/ml). Potassium was at 3.3 mmol/L (normal value, 3.5-5.5 mmol/L) with serum calcium of 1.94 mmol/L (normal value, 2.1-2.5 mmol/L) at presentation. Other investigation at baseline revealed high serum lactate (6.6 mmol/L), deranged renal function (urea, 32.8 mmol/L; serum creatinine, 216 umol/L), elevated bilirubin (78 micromol/L), and mildly elevated liver enzymes (ALT, 67; AST, 154). He was also anaemic with leukocytosis and thrombocytopaenic at presentation (Hb, 10.7 g/dl; total white cells, 11.4 × 10^9^/mm^3^ and platelet 20 × 10^9^/mm^3,^ respectively). Of note, a blood film was sent for malaria parasite detection from the ED as the travel history might have prompted this, as this is not routinely carried out in all the febrile cases presenting to our ED unit.

A clinical diagnosis of AMI was made in the ED and the patient was promptly referred to a cardiologist. Bedside echocardiogram performed and showed hyperdynamic contractility with preserved LV systolic function. Inotrope and oral statin were commenced and urgent coronary angiography was performed, as thrombolysis was contra-indicated due to his marked thrombocytopaenia. Angiography revealed normal coronary arteries and left ventriculogram. The diagnosis of AMI was revised, as the repeated cardiac enzymes at least six hour apart were also negative. Later, on further questioning, he revealed that he had been working in Gabon for six weeks, returning three weeks prior to his presentation. He had received yellow fever immunization but had not been advised to take any anti-malarial chemoprophylaxis prior to travelling. A week after his return to Malaysia, he developed fever with myalgia but no chills or rigors. As the symptoms were not severe, he then flew to Hat Yai, Thailand for a vacation. He stayed there for four days before returning to Malaysia. With this history revealed, his blood film for malaria parasite was traced and found to be positive 24 hours later.

On day 2 of admission, he was found to be tachypnoeic, hypotensive, pale, and jaundiced. He remained afebrile and examination of the other systems remained the same. Identification of the malaria parasite was done using Binaxnow® Malaria (Binax, Inc, Portland, ME, USA) rapid diagnostic test and confirmed to be *Plasmodium falciparum* via a real time PCR-based test [1]. The parasite burden was 6%. The diagnosis was revised to severe malaria and intravenous artesunate and oral doxycycline were promptly initiated. He was promptly transferred to the intensive care unit (ICU).

Unfortunately, a few hours after ICU admission he developed progressive hypoxemia due to acute respiratory distress syndrome (ARDS) which necessitated invasive ventilation. He underwent continuous veno-venous haemodialysis (CVVHD) due to oliguric acute kidney injury with a peak urea of 14.3 mmol/L and creatinine of 450 micromol/L with noradrenaline support. Intravenous ceftriaxone was added to cover for possible super-added bacterial infection. Normoglycaemia was maintained throughout his stay in ICU with intravenous 5% dextrose solution. After six hours of commencement of the first dose of IV artesunate, the parasite count rapidly declined to 0.07%. Following anti-malarial therapy, the inotrope requirement was gradually reduced. The parasite count was undetectable after 48 hours of IV artesunate.

He was extubated after six days in ICU. His renal function and urine output continued to improve and he eventually did not need renal replacement therapy. On discharge, his urea and creatinine were stable at 7.6 mmol/L and 200 umol/L, respectively. The antibiotics were ceased after ten days as baseline and subsequent blood cultures did not grow any bacteria. He received in total ten days of weight-adjusted doses of IV artesunate, followed by four doses of fixed combination pills of artemisinin and lumefantrine and 14 days of oral doxyxycline. A month later on follow up, his urea and creatinine normalized to 3.5 mmol/L and 85 umol/L, respectively.

## Discussion

Malaria is a serious and potentially life-threatening disease, especially so in travellers due to: 1) lack of protective immunity to the parasite and, 2) due to delay in recognizing and treating the infection upon return. The clinical presentation of malaria is non-specific and is often misdiagnosed [2]. Of note cardiac involvement is a recognized manifestation of malaria. This includes conduction abnormalities, MI and non-specific ST-T segment changes [3]. In a study done amongst 161 cases of *P. falciparum* infections, 23 patients (14.3%) had either non-specific T-segment changes or abnormal conduction delay [4]. Interestingly in this cohort, despite aforementioned changes, the cardiac biomarkers were rarely elevated (inclusive of CK, CKMB and troponin I).

Transient myocardial ischemia manifested by ST segment changes may not cause elevation in the cardiac biomarkers. This might be due to diffuse distal vessel epicardial spasm and microvascular spasm [5]. This is termed coronary microvascular dysfunction characterized by angina pectoris and non-significant obstructive coronary atherosclerosis [6]. The link between this and Plasmodium infection can be related to *Plasmodium falciparum* ability to produce distinctive virulence factors that increase the risk for the human host to develop end organ complications. It increases microvascular sequestration via the expression of several molecules on the infected erythrocyte such as Ag332 [7], modified band 3 [8] and *P. falciparum* erythrocyte membrane protein [9]. This may be the same pathophysiology of myocardial ischaemia in malaria infection.

Of note, hypokalaemia and hypercalcaemia which can mimic the ST-T changes of myocardial infarction was not present in this patient on admission. [10].

## Conclusion

This case report illustrates that diagnosis of malaria based on clinical features alone is difficult and requires a high index of suspicion among clinicians. The key to diagnosing malaria is to elicit detailed travel history and request a specific malaria test in anyone who feels unwell and has recently been to an endemic country. The diagnosis of acute coronary syndrome due to severe malaria should be considered in those who present with features suggestive of myocardial ischaemia.

All travelers to malarious areas should be made aware of the risk of contracting malaria and the seriousness of this infection. They should be advised on preventive measures, which include complying with anti-malarial chemoprophylaxis and avoiding mosquito bites. Furthermore, patients need to be informed about the importance of seeking medical treatment immediately should they become ill during travel or up to one year after returning home.

## Consent

Written informed consent was obtained from the patient for publication of this case report and any accompanying images. A copy of the written consent is available for the review by the editor of this journal.
